# Bipolar disorder dynamics: affective instabilities, relaxation oscillations and noise

**DOI:** 10.1098/rsif.2015.0670

**Published:** 2015-11-06

**Authors:** Michael B. Bonsall, John R. Geddes, Guy M. Goodwin, Emily A. Holmes

**Affiliations:** 1 Department of Zoology, University of Oxford, Oxford OX1 3PS, UK; 2 St Peter's College, Oxford OX1 2DL, UK; 3 Department of Psychiatry, Warneford Hospital, University of Oxford, Oxford OX1 7JX, UK; 4 Medical Research Council Cognition and Brain Sciences Unit, Cambridge CB2 7EF, UK; 5 Department for Clinical Neuroscience, Karolinska Institutet, Stockholm, Sweden

**Keywords:** likelihood, mechacognition, mood variability, mood dynamics

## Abstract

Bipolar disorder is a chronic, recurrent mental illness characterized by extreme episodes of depressed and manic mood, interspersed with less severe but highly variable mood fluctuations. Here, we develop a novel mathematical approach for exploring the dynamics of bipolar disorder. We investigate how the dynamics of subjective experience of mood in bipolar disorder can be understood using a relaxation oscillator (RO) framework and test the model against mood time-series fluctuations from a set of individuals with bipolar disorder. We show that variable mood fluctuations in individuals diagnosed with bipolar disorder can be driven by the coupled effects of deterministic dynamics (captured by ROs) and noise. Using a statistical likelihood-based approach, we show that, in general, mood dynamics are described by two independent ROs with differing levels of endogenous variability among individuals. We suggest that this sort of nonlinear approach to bipolar disorder has neurobiological, cognitive and clinical implications for understanding this mental illness through a mechacognitive framework.

## 1. Introduction

Bipolar disorder is a chronic recurrent mental illness [1]. The global health burden of bipolar disorder is dramatic: 1–4% of adults live with the condition and current estimates suggest that this mental illness accounts for up to 10% of the burden of all mental and substance use disorders (in terms of years lived with the disability—YLDs) and that this equates to about 17.5 million YLDs [2]. Bipolar disorder is characterized by pathological mood instability including episodes of both extreme low (depressed) mood and elevated (manic) mood, interspersed with less severe but still problematic mood fluctuations or, in some people, relative mood stability [3]. Disproportionate numbers of teenagers and young adults (15–24 years) are diagnosed with the disorder (compared to other mental and/or substance use illnesses) [4]. Younger age of disease onset is associated with higher suicidal risk, with lifetime suicide attempt rates estimated between 20 and 47% [5] and as such bipolar disorder has the highest rate of suicide across all psychiatric disorders [6]. The neurobiology of bipolar disorder is poorly understood, and standard treatment involves long-term pharmacological interventions often with poor clinical responses [3,7]. There have been no significant clinical advances since the use of lithium in the 1950s. A better understanding of processes underlying the disorder is required.

There has been a traditional emphasis on extreme mood episodes both in defining the disorder and developing treatments. This approach has underestimated the inter-episode morbidity and ignored mood variability as a key feature of bipolar disorder. We have previously argued that mood variability provides a potential focus both for understanding the dynamic interactions that occur between mood elevation, depression, anxiety and the environment [8] and that such an understanding is important for experimental manipulations with medicines or psychological treatments [9]. Our understanding of this complex and dynamic temporal mood variability in bipolar disorder may be advanced by the use of quantitative analytic methods such as those developed through the application of nonlinear dynamics to physical, social and biological systems (e.g. [10]).

Descriptive patterns of the dynamics of bipolar disorder [9,11] reveal that simple time-series approaches may provide a robust way to characterize and corroborate clinical judgement on bipolar disorder mood stability. However, a more mechanistic theory for bipolar disorder is clearly warranted in order to develop a more deductive approach to developing testable hypotheses rather than simply describing patterns.

As noted, bipolar disorder is conventionally characterized by depressive and manic episodes although inter-episodic mood instability is increasingly recognized. This variability in mood provides a basis for developing mathematical approaches based on limit cycle oscillators [12–15] to understand the dynamics of this mental health disorder. However, the depressive and/or manic symptoms do not seem to be simple oscillatory swings between two states of ‘mania’ and ‘depression’, and the functional outcome of the disorder is not simply related to recovery from the acute changes in mood but also to the high levels of inter-episodic mood instability [16–18]. In particular, our recent analyses of mood instabilities in 23 individuals [9] highlighted that dynamics above and below a threshold of ‘average’ levels of mood are described differently. To build on this initial work, in this study we develop a mechanistic framework to understanding the dynamics of bipolar disorder using relaxation oscillators (ROs).

**Figure 1.**
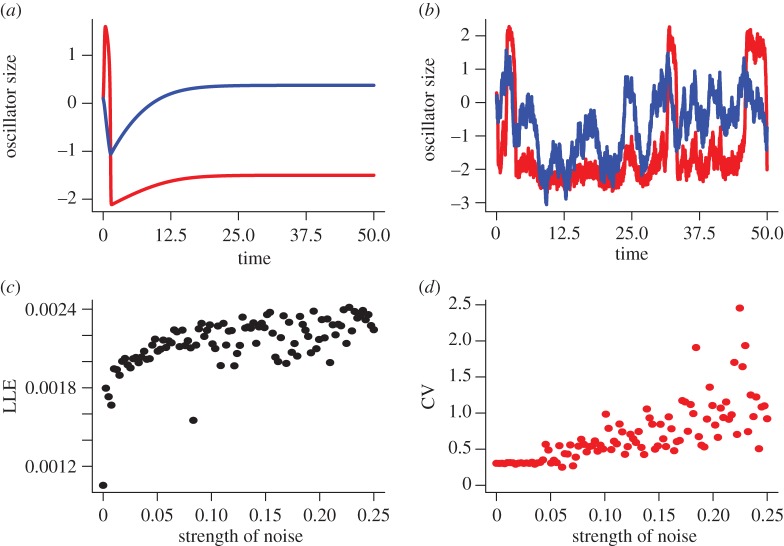
Analysis of a single oscillator in the presence of noise. (*a*) When noise is absent and |*a*| > 1 the system (equations (2.1) and (2.2)) is stable. Increasing levels of noise lead to (*b*) increasing tendency for fluctuations in oscillators. This is characterized by increases in both (*c*) Lyapunov exponents (LLE) and (*d*) coefficients of variation (CV) as the strength of the noise increases (Parameters *a* = 1.5, *b* = 30).

ROs comprise a class of nonlinear periodic dynamic systems. Importantly, such cyclic systems are characterized on different timescales; intervals of time during which there is little change in state are interspersed with rapid periods of change in state (e.g. [19]; figure 1). In comparison to other mathematical approaches modelling bipolar disorder, ROs provide a relevant context in which to describe ongoing changes in mood state—periods of high and/or low mood are interspersed with periods of relative mood stability or instability [20,21].

If multiple oscillatory processes operate at the molecular level in bipolar disorder, modelling the interactions of ROs at higher levels of organization (such as the mood level) might provide additional insight into the dynamics of bipolar disorder within and among individual patients. That is, by using mathematical approaches to move down a hierarchy from mood fluctuations to candidate, molecular processes may allow insights through a mechanistic approach to cognition which will be generally necessary for thoroughly comprehending mental illnesses [22]. This *mechacognitive approach* may, by developing descriptions of affective instabilities, lead to novel approaches to understand the underlying biological instabilities.

Characterizing coupling in the nonlinear dynamics of mood also necessitates a thorough consideration of noise. In dynamical systems, stochastic fluctuation or variability is an integral component of the dynamics and can be generated by internal fluctuations or by external perturbations. Internal fluctuations arise through the stochastic sampling of the parameters and processes driving the deterministic dynamics; external stochastic effects operate to affect the change in mood from one time point to the next. The effects of noise have been well explored in other contexts (e.g. [23–25]) and often involve establishing the most appropriate way in which to combine both variability and deterministic dynamics. In understanding bipolar disorder dynamics, mood fluctuations have been characterized as noisy and nonlinear [9,26]. Understanding the interplay between stochastic fluctuations and deterministic mood signal requires greater and more detailed scrutiny of the dynamics associated with individual mood profiles.

Here, we investigate how the dynamics of subjective experience of depressed mood (the dominant mood state) in bipolar disorder can be understood using a RO framework and test the model against mood time-series fluctuations from a series of individual participants who reported their mood symptoms weekly using a standardized measure of depressed mood. We begin by outlining the mathematical model and analysis of the model. Building on these results, we show how the dynamics of ROs can be linked to empirical patterns in individual participant mood fluctuations. We show how deterministic patterns and stochastic volatility vary among individuals and we discuss the results in the light of recent advances in understanding the dynamics of non-communicable dynamical diseases such as bipolar disorder.

## 2. Methods: mathematical model and time-series analysis

We use a RO framework to explore the dynamics of bipolar disorder [27]. The model shows a range of dynamics and, as a set of ordinary differential equations, is of the general form:2.1
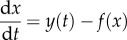
and2.2
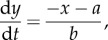
where *x* and *y* are state variables, *a* and *b* are (unknown) parameters and *f*(*x*) is of the form −*x* − (*x*^3^/3). This oscillator (equations (2.1) and (2.2)) is a based on a van der Pol type RO [19,27] and in the absence of noise and coupling has the following fixed points:2.3
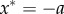
and2.4
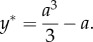


Linear stability analysis of this RO reveals that the eigenvalues are 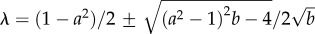 and (local) stability holds if as *b* → 0, *a* > 1.

### 2.1. Mood model formulation

To explore how this RO can be used to understand patterns of mood fluctuations, we begin by assuming that mood fluctuations through time (*t*) are assumed to be an unknown function of at least two processes (**X**) and (**Z**) (tacitly we might assume that these processes might represent states of high and low mood). If average mood (*M*) varies through time such that:2.5
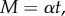
and mood is related to the first process (**X**) by:2.6
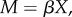
and the second process (**Z**) by:2.7
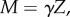
then the overall changes in mood through time can be represented by a total derivative:2.8
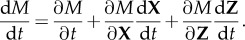
So from equations (2.5)–(2.7):2.9
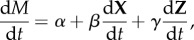
where **X** and **Z** represent (in vector form) the ordinary differential equations (equations (2.1) and (2.2)) for two independent oscillators.

Additionally, oscillators are likely to be coupled and noisy. Extending equations (2.1) and (2.2), the ordinary differential equations for the *i*th oscillator follow the general dynamics:2.10
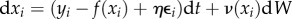
and2.11
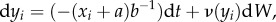
where *x_i_* and *y_i_* are state variables. As defined for equations (2.1) and (2.2), 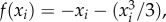 and *a* and *b* are parameters. *η* is a coupling weighting and for 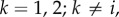 coupling strength (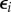) is 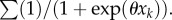 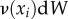 and 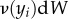 are noise terms acting on the *i*th RO.

### 2.2. Bipolar time-series data

Forty-two participants (table 1) with bipolar disorder from a local mood disorders clinic were recruited for this study—further demographic and clinical details are given in table 1. All participants gave their written informed consent and completed standardized questionnaires for baseline assessment; these assessments measured levels of depression (QIDS-SR16 (Quick Inventory of Depressive Symptomatology); [28]), mania (Altman self-rating scale for mania; [29]) and trait anxiety (Spielberger state anxiety inventory; [30]) (table 1).

**Table 1.** Socio-demographic and clinical characteristics of participants for the AR and RO analysis. The demographic and clinical statistics are only given for the majority (19/25) of participants where two independent ROs were needed to describe their mood fluctuations—see Results for further explanation.

	AR analysis (*n* = 42)		RO analysis (*n* = 19)	
characteristics	mean	s.d.	mean	s.d.
age at study intake, years	41.3	11.8	40.2	13.2
age at illness onset, years	20.4	8.7	20.9	11.8
duration of illness, years	21.0	11.8	19.9	14.0

	number	%	number	%
male	11	26.2	1	5.3
female	31	73.8	18	94.7
ethnic origin				
mixed	2	4.8	1	5.3
White	38	90.5	17	89.5
Chinese	1	2.4	1	2.4
Asian	1	2.4	0	0
educational level, years				
without diploma equivalent	1	2.4	0	0
high school graduate	7	16.7	4	21.1
college graduate	33	78.6	15	78.9
unrecorded	1	2.4	0	0
bipolar I disorder	29	69.0	15	78.9
bipolar II disorder	13	31.0	4	21.1

The present analysis focuses on depression ratings measured with the QIDS-SR16 scale. QIDS-SR16 consists of a 16-item questionnaire measuring severity of depression, covering the nine DSM-5 major depressive disorder symptoms (e.g. sadness, loss of pleasure and weight change). Participants were asked to choose the response that best described themselves over the past 7 days on a four-point scale (0–3) anchored at all points by a description. For example, Question 5, ‘feeling sad’ is anchored at 0 = ‘I do not feel sad’, 1 = ‘I feel sad less than half the time’, 2 = ‘I feel sad more than half the time’ and 3 = ‘I feel sad nearly all the time’. While scores on the QIDS-SR16 can be clinically grouped into five severity levels: none (0–5), mild (6–10), moderate (11–15), severe (16–20) and very severe (21–27), here we consider that scores vary continuously across this scale. The QIDS-SR16 has established psychometric properties for rating depressive symptom severity in individuals with bipolar disorder as well as chronic major depressive disorder. This rating system is known to be strongly (positively) correlated with clinician-reported scales [28,31].

Weekly mood score data on the QIDS-SR16 were collected through True Colours (https://truecolours.nhs.uk/www/), a system using SMS (cell phone short messaging service) and e-mail/Web interfaces developed in a local mood disorders clinic [21,32] to capture individual reported outcome measures. Participants were given credit card-sized versions of the QIDS-SR16 scale to carry in daily life and consult when making ratings. A bespoke computer program automatically sent out weekly text messages to participants' cell phones to prompt them to submit their self-rating. To do this, participants simply replied with a text message containing a list of numbers corresponding to their self-rating on each of the QIDS-SR16 items.

Infrequently, participants replied with more than one text per week. The first valid response to the weekly prompt was used in the analysis, and subsequent responses within the week removed. If no valid response was received within the week following the prompt, this was coded as missing data up until an individual's last actual response to the system. In addition, the SMS system occasionally did not send prompts (e.g. if a participant requested, temporarily, to take a break from texting). Weeks in which no prompt was sent were also coded as missing data.

### 2.3. Time-series analysis

We investigate the descriptive properties of the individual participant time series using autoregressive (AR) and threshold AR statistical models (following Bonsall *et al*. [9]) and use results from RO analysis (see above) to explore the nonlinear dynamics of mood variability in individuals.

### 2.4. Likelihood framework

Based on our previous analysis, the distribution of mood scores is well characterized by a gamma distribution [9]. This probability distribution function is described by two parameters: a rate (*r*) and a shape (*s*) parameter:2.12
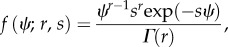
where *ψ* is the observation and *Γ*(*r*) is a gamma function (*Γ*(*r*) = (*r* − 1)!). The expectation from this distribution is *μ* = *r*/*s* and the variance is *σ*^2^ = *r*/*s*^2^. From a time series of *N* mood scores, we use this probability distribution function to construct an appropriate likelihood *L* where the first point in the series is conditioned on the mean and subsequent points on the previous observation:2.13
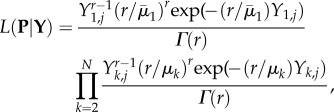
where **P** is an unknown parameter set, **Y** is a set of observations, 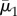 is the mean of the *j*th series and *Y_k_*_,*j*_ is the *k*th mood score (out of *N*) for the *j*th participant. *r* is an additional (nuisance) parameter associated with the likelihood function. *μ_k_* is the expected mood score for the *k*th time point derived from the underlying dynamical model (equation (2.9)). This expected mood score at each time point (*k*) is determined by numerically integrating the model over the weekly census interval (*T*) of the participant's mood time series:2.14
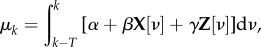
where **X** and **Z** represent two different oscillators (which may be coupled, e.g. equations (2.10) and (2.11)). We fit this likelihood to each participant's mood time series. Computationally, to minimize the negative log-likelihood, we use an expectation-maximization method to deal with missing values within a modified simplex algorithm [9]. Goodness of fit between model predictions and mood observations is evaluated through the use of Akaike information criterion (AIC) scores (a penalized likelihood metric based on the magnitude of the likelihood and the number of estimated parameters: 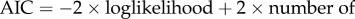 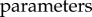
—with lower AICs giving greater concordance between model and data) [33] and measures of RMSEs (the square-root of the mean squared difference between an observed data point and model prediction for each time point: 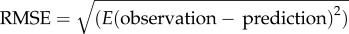).

## 3. Results

### 3.1. Relaxation oscillators

In this section, we investigate the dynamical patterns expected with noisy, coupled oscillators. As noted, the RO (equations (2.1) and (2.2)) is stable if as *b* → 0, *a* > 1. In figure 1*a*, we illustrate that stable dynamics (when *a* = 1.5). Noise-induced instabilities give rise to fluctuations in the oscillators (figure 1*b–d*). Characterization of the local Lyapunov exponents for stochastic systems [34] illustrate that close-by trajectories diverge with increasing levels of noise and there is a critical noise threshold (determined from estimates of the coefficient of variation in the magnitude of the fluctuations) beyond which the system has an increased tendency to fluctuate.

**Figure 2.**
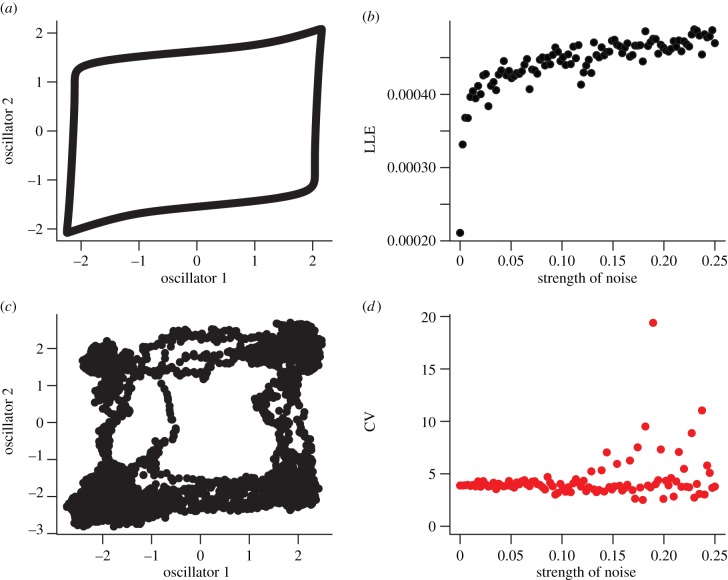
Analysis of two-coupled oscillators in the presence of noise. (*a*) In the absence of noise, coupled oscillators show regular fluctuations and are in phase at coordinates (−2,−2) and (2,2). In the presence of increasing levels of noise (*c*), this regular trajectory is disrupted and there is more *time* where oscillators are in phase. Unstable fluctuations are characterized by increases in both the (*b*) Lyapunov exponent (LLE) and (*d*) coefficients of variation (CV) with increasing noise levels (Parameters *a* = 1.5, *b* = 20, *e* = 0.5).

Increasing levels of coupling in the presence of noise gives rise to increasing tendencies for the system to oscillate and also affects the degree of synchrony observed between ROs: increases in the noise increases the degree of synchrony between coupled ROs (figure 2).

### 3.2. Descriptive time-series models

**Figure 3.**
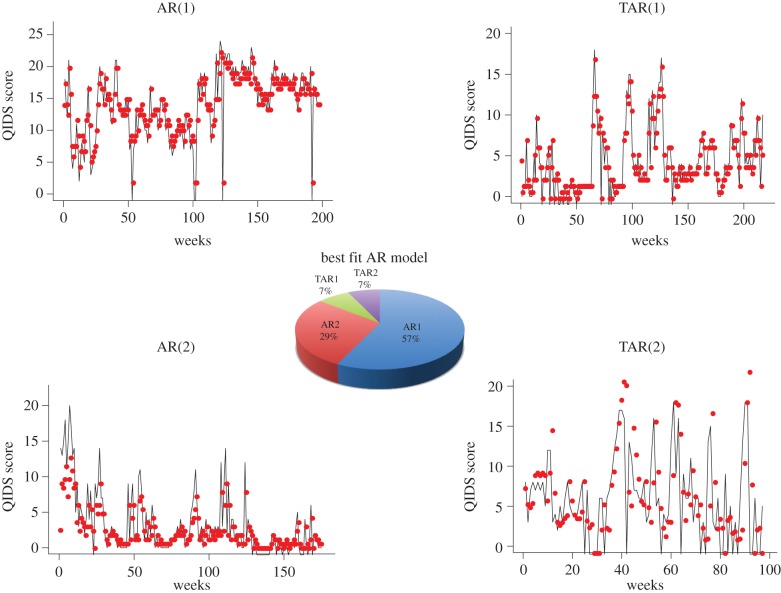
Descriptive time-series analysis using AR models. From 42 individual time series, over half (57%) are described by single lag AR process. One-third (29%) are described by two lag AR process. Threshold AR processes (which described different dynamics above and below average model score) describe 14% of the time series. Representative fits of each of the AR processes (red points based on one-step ahead predictions) to the mood fluctuation time series (solid black lines) are shown.

To test the hypothesis that mood dynamics are noisy and nonlinear, we fit linear and threshold AR models to the individual (42 participants with bipolar disorder) time series. Based on AIC scores, the overall best-fitting model was the AR(1) model (57% of time series). The other models, AR(2), TAR(1) and TAR(2) are best fits for 29%, 7% and 7% of the time series, respectively. One-step ahead predictions and goodness of fit of these models to individual participant time series are shown in figure 3.

### 3.3. Mechanistic bipolar disorder models

To explore the correlative patterns identified by this preliminary, descriptive time-series analysis, we fit dynamic ROs to a subset of the individual time series. Based on 25 participants (with a range of time-series lengths (79–233 weeks) and proportion missing values (0–0.44)), the RO analysis revealed (based on AIC rankings) three individual time series described by a single RO, three individual time series described by two deterministically coupled ROs and the majority of mood time series (19) described by two independent ROs.

**Figure 4.**
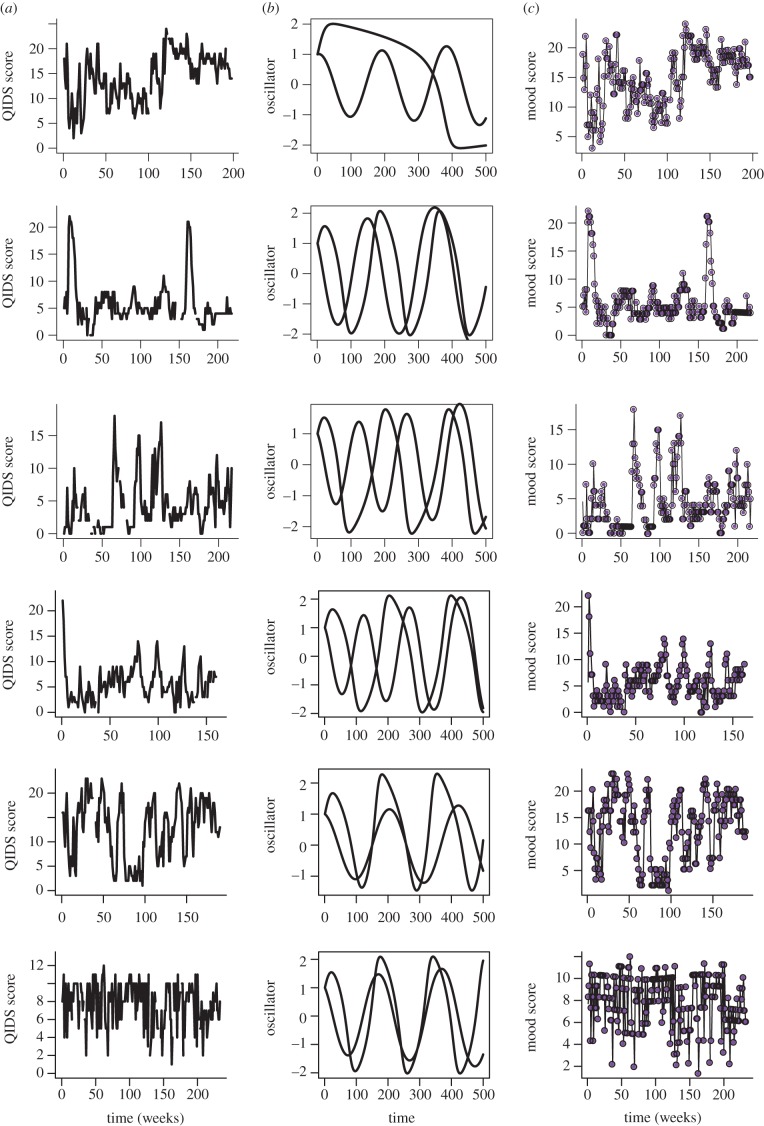
Illustration of predicted RO dynamics. Observed mood fluctuations (QIDS scores), predicted RO dynamics and predicted fit to QIDS scores for six participants (whose dynamics are described by two independent ROs). (*a*) Time series of observed mood dynamics (QIDS scores). (*b*) Predicted RO dynamics from numerically integrating equations (2.1) and (2.2). Parameters *a* and *b* are derived from the maximum-likelihood fitting of equation (2.9) to the observed mood dynamics. (*c*) Predicted mood dynamics (from the total derivative (equation (2.9))) determined by using each observed QIDS score to predict the next mood score. Again, parameters (*α*, *β* and *γ*) are derived from the maximum-likelihood fit of equation (2.9) to the overall observed mood dynamics.

Focusing on these 19 mood time series described by two independent ROs, the predicted dynamics for the two independent ROs for participant mood time series are shown in figure 4. Using model predictions (based on using a current QIDS observation and the parametrized total derivative to predicted the next observations), these ROs together with average mood levels and strength of oscillators predict the observed QIDS time series (figure 4*a*–*c*).

**Figure 5.**
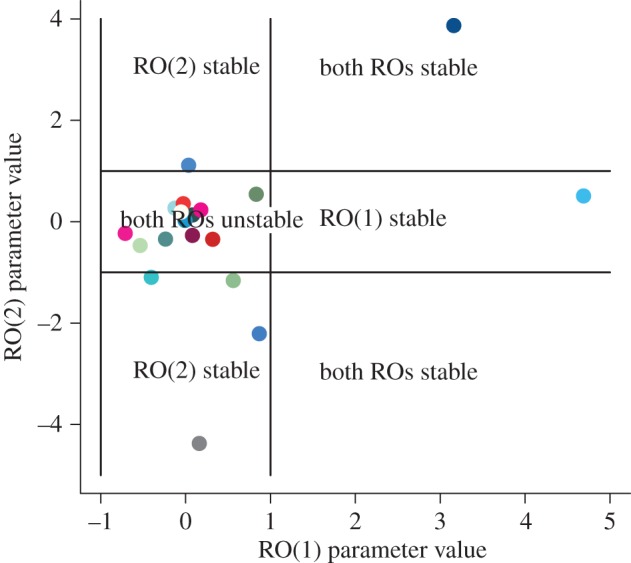
Goodness of fit of RO dynamics to a subset (25) individual time series. Model selection shows that the dynamics are best described by independent ROs. Nineteen out of 25 participants have dynamics described by independent oscillators. Based on local stability analysis when |*a*| > 1, the dynamics are stable, and of these 19, 12 participants have time series best explained by unstable (oscillating) ROs. (Each colour corresponds to a different participant's time series and we use the same colour for a participant's time series across figures 5–7.)

In the absence of noise, from the stability analysis of independent ROs, the expected dynamics of each oscillator are dependent on the magnitude of a key parameter (*a*). Again, focusing on the 19 mood series identified to be described by two independent ROs, figure 5 shows regions of the expected deterministic dynamics (in the absence of noise) of the independent ROs (stable and unstable) based on statistical estimates of this key parameter (*a* from equations (2.1) and (2.2)). The dynamics, as noted, are expected to reach a stable point if in both |*a*| > 1. In 12 out of 19 (2/3) of these mood time series, the underlying deterministic dynamics of each of the independent ROs are expected to oscillate. In six out of 19 (1/3), one RO is expected to be stable while the other cycles. In one case (1/19), both independent ROs are expected to be stable (figure 5).

**Figure 6.**
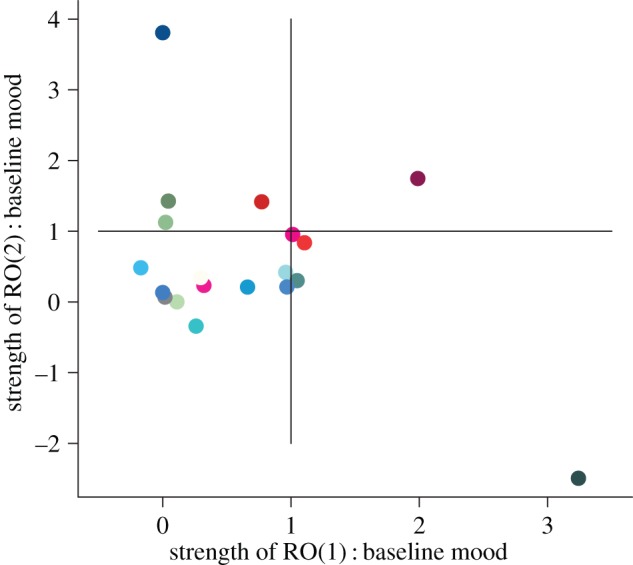
Contribution of the ROs to mood fluctuations. Based on the total derivative (equation (2.9)), the strength of each oscillator is determined by a parameter (*β* or *γ*). The ‘average’ mood level is determined by parameter *α*. We evaluate the ratio between these parameters to determine whether the ‘average’ mood level or the oscillator is the predominant driver. If the ratios (*β* : *α* and *γ* : *α*) are less than 1, then average mood predominates over the RO dynamics. In 12 out of 19 participants (represented by coloured points), both ratios (*β* : *α* and *γ* : *α*) are less than 1. If the ratio is greater than 1, then oscillating dynamics are the predominant drivers of mood variability. ROs are the predominant drivers of mood variability in seven out 19 participants. (Each colour corresponds to a different participant's time series and we use the same colour for a participant's time series across figures 5–7.)

The total derivative (equation (2.9)) shows that changes in mood through time are a function of both average mood (*α*) and the changes that are driven by the strength of the ROs (*β*(d**X**/d*t*), *γ*(d**Z**/d*t*)). To evaluate the importance of average mood versus the RO, we compute ratios of magnitudes of effect for RO versus average mood (*β* : *α* and *γ* : *α*). Figure 6 shows these relative effects of average mood (*α*) to that of the effect of the ROs (*β* : *α*, *γ* : *α*) on overall mood dynamics for the 19 mood series identified to be described by two independent ROs. In the majority of cases (12/19), average mood is the predominant driver of mood variability. In these cases, as illustrated in figure 6, the ratios of *β* : *α* and *γ* : *α* are both less than one. ROs are the major driver of mood dynamics in seven out of 19 cases. In six of these cases, only one of the ROs predominates in driving the mood fluctuations (either the ratio *β* : *α* or *γ* : *α* is greater one), while in a single case, both ROs drive mood dynamics (the ratios of *β* : *α* and *γ* : *α* are both greater than one).

**Figure 7.**
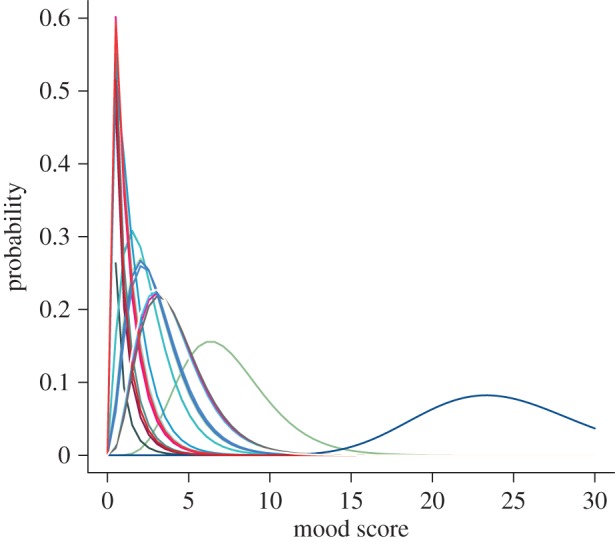
Relative variance in mood (QIDS) score for 25 participants from time-series analysis. Based on the underlying gamma likelihood, the shape of the underlying probability distribution shows the contribution of variance (noise) to fluctuations in observed dynamics. Each colour represents an individual participant's probability distribution of QIDS scores. (Each colour corresponds to a different participant's time series and we use the same colour for a participant's time series across figures 5–7.)

Furthermore, the shape parameter (*s*) from the gamma likelihoods (see Methods) for the best-fitting RO to each time series reveals that relative variability in mood between the participants to be large (figure 7). The observed variability in shape parameters suggests that in addition to the deterministic dynamics driven by the ROs, mood fluctuations for each participant are subject to stochastic volatility. From the range of probability distributions observed in figure 7, some individual time series are subject to much more variability than others and this has implications for characterizing the mood profile dynamics and broader clinical implications.

## 4. Discussion

Here, we have shown that mood fluctuations in bipolar disorder are driven by coupled effects of deterministic dynamics (captured by ROs) and stochastic variability. Using an appropriate likelihood framework, we show that, in general, mood dynamics are described by two independent ROs. Moreover, individual average levels of depression and stochasticity (variability) are also important and essential drivers of mood dynamics and fluctuations. As this variability is an integral part to these dynamics, transitions between states driven by stochasticity [35] have implications for predictive limits in these sorts of nonlinear systems.

However, details matter. Both qualitative and quantitative differences among individual participants highlight the drivers of mood variation and fluctuations. In contrast to recent suggestions that mental illnesses such as depression can be captured with simple time-series metrics (e.g. measures of the degree of autocorrelation and/or coefficient of variation) indicative of critical slowing down [35–37], we argue that bipolar disorder dynamics are better explained by developing appropriate dynamical models coupled to robust statistical methods of analysis. As noted, relaxation oscillations predict long periods of dynamics in a (low or high) state with a rapid switch to the alternative state. It is unlikely that these dynamical changes will be captured with simple time-series statistical metrics. Furthermore, statistical metrics are unlikely to be sufficiently robust or indicative of the sorts of drivers of alternating dynamical states. We argue that more work on mechanistic modelling approaches such as those we develop here and elsewhere [9] are clearly warranted. This is necessary as idiosyncratic (individual-level) patterns give rise to classes of dynamics of mood variation and hence a wide range of affective instabilities (figures 4–7).

ROs have been widely applied to problems across a range of specific disciplines including cell biology (e.g. [38]), neurosciences (e.g. [39]), cardiology (e.g. [40]) as well as synthetic biology (e.g. [41]). We have focused on the idea that an oscillator describes mood dynamic departures from an average state through relaxation to an episodic state and that these oscillators could be coupled either directly or indirectly through noise. Coupling oscillators has implications for better understanding the biological or psychological and even social rhythms of bipolar disorder (e.g. [42]), and this is exactly what we need to be better able to measure mood to make improved clinical progress.

Originally, Winfree [43] argued that oscillators that interact *weakly*, either deterministically [44] or through correlated noise effects [45], can generate rhythmic patterns in biological systems and this can influence the dynamical state between oscillators (e.g. degree of synchrony). As the variation or difference among oscillators gradually reduces, there is a critical transition past which dynamics are identical (e.g. synchronous). Knowing this coupling effect or its drivers is likely to have important implications for mental illnesses such as bipolar disorder. If, as the data suggest, the general condition is that noisy ROs capture mood dynamics then it is natural to ask about the number of oscillators and their overlap and/or strength of coupling, needed to influence dynamical outcomes [44]. More importantly, from a clinical perspective, will be whether the degree of critical coupling strength has implications for treatment effects and prospective predictions on disease progression. Alterations in the amplitude and/or phase of the oscillators could have important consequences for understanding the dynamical instabilities that drive bipolar disorder episodes (see below: information flow). Further work should aim to identify the drivers of these instabilities (at the various levels of mechanism including both the molecular and behavioural scales) as it could allow clinicians to both assess and track changes in mood patterns underlying bipolar disorder episodes, and allow a better test of response to treatment or much needed treatment innovation.

Using theory to explain affective instability phenomena has parallels with classic theory on the spread of communicable diseases such as measles, cholera or malaria. In classic epidemiology, the specific details of any particular disease are collapsed (at the population level) into classes of susceptible, infected and recovered individuals, and key mathematical parameters (e.g. [46]). A key parameter in this epidemiological theory is the disease transmission rate: how effective is the disease at spreading. This parameter subsumes many of the intricacies and details of disease biology. Yet, this sort of theory has proved successful for understanding general and specific disease dynamics, spread and control [46]. Similar principles can also be applied to the theory we develop here for the dynamics of non-communicable mental illnesses (such as bipolar disorder). For instance, the importance of mean levels of depression and the influence of oscillators) coupled to endogenous variability, may turn out to be of real practical significance as a treatment target. Currently, prevention of relapse is the objective of long-term treatment. Average inter-episode sub-syndromal symptom levels are not routinely a key target of treatment. The models reported here predict that average levels of mood have a major impact on longer-term mood stability and this is consistent with the finding (in the wider cohort of participants) that mean depression ratings predict direct treatment costs in a cost of illness study.

The precise physical or biochemical equivalents of the ROs are of course not established by studies of this kind. Indeed, we have limited mechanistic insight into the specific neurobiological drivers of mental disorders at the level of neurons, within-brain or cortex dynamics that lead to the manifestations of disease. However, alterations in physiology (e.g. decreased pH levels) and modification of mitochondrial activity [47–49] have been hypothesized to be associated with episodes of depression [47]. Molecular genetic studies have also implicated genes such as CACNA1c [50] encoding calcium channels and other pathways that impact Ca^2+^ regulation and affect individual metabolism and activity [51,52]. Alterations in calcium metabolism are predicted to affect mania and rapid cycling in bipolar disorder [47]. Recently, mathematical modelling approaches have been developed to investigate these more causal links between the biological mechanism and fluctuations of bipolar disorder [14,15]. Goldbeter [15], using sets of coupled ordinary differential equations, explored how inhibition and feedbacks can drive bistability, oscillations and higher-order nonlinear dynamics in bipolar disorder. The critical aspect of this work by Goldbeter [15] was to link the mutual drivers of inhibition (described by Hill functions) to propensities for mania and depression. This work has strong parallels with our study. Although, we adopt a broader mechanistic approach, our RO model (equation (2.9)) has the propensity to show a range of dynamics that can be (putatively) linked to the biological mechanism and we use mood fluctuations to characterize model parameters and hence dynamics. Constructing relevant mathematical approaches that link different scales (e.g. molecules to neurons to behaviour) through a mechacognitive understanding may be a fruitful approach in understanding the dynamics of mental disorders.

Affective instability [53] is seen in a range of psychiatric conditions. Indeed, it has been argued that affective instability is a trait [53] that, as in our case, leads to significant fluctuations in mood (without necessarily leading to the full blown episodes of depression and/or mania as in bipolar disorder). Marwaha *et al*. [53] informally identified four components of affective instability: (i) the rapid oscillation and intensity of affect, (ii) a capacity to control affect, (iii) its behavioural consequences, and (iv) triggers that stimulate affect change. As developed in our time-series studies ([9] and here), it is important to operationalize the measurement of these attributes. We have obviously explored the first: rapid oscillation and intensity of affect. This provides a framework to examine the generality of this feature across different clinical conditions and its status as a trait. Using a novel statistical framework for the mechanism associated with bipolar disorder dynamics, we develop an approach for integrating hierarchical information flow [54] within the paradigm of mechacognition. By describing mood dynamics in terms of coupled ROs, we build mechanism into the understanding of (cognitive) mood variability. This differs from the descriptive approaches used elsewhere (e.g. [9,37]) that might be argued useful in characterizing affective instabilities in terms of the phenomenology of mood fluctuations.

In summary, we have proposed a framework for linking across scales of organization associated with bipolar disorder dynamics and, more broadly, affective instability. We develop an approach for integrating hierarchical information flow [54] within the paradigm of ‘mechacognition’. By describing mood dynamics in terms of coupled ROs, this extends the descriptive approaches used elsewhere (e.g. [9,37]). As Rabinovich *et al*. [54] further highlighted the hierarchy of interactions and how dynamics within the brain (at the neuron level) scale-up to influence broad cortex dynamics requires a greater understanding of information flow across different scales of organization [54]. Disruption and disturbances to this information flow are predicted to lead to cognitive disorders (such as in bipolar disorder). Developing an appropriate framework is required to describe information flow up and down the ladder of mechanistic cognition (from neuroscience, psychology and pharmacology and beyond) and is likely to help fuel innovation and novel clinical treatments. While the ideal of individualized treatments [55] is an aspirational goal, a more pragmatic path might be to develop novel hierarchical approaches to understanding mood disorders [56]. Our insights from repeated mood monitoring, and the sort of preliminary analyses of the data that we have completed here, opens the possibility that linking accurate phenotypic measurement with genes, neural activity, physiology and cognition within a mathematical framework might be a fruitful approach in both understanding the neurobiology and the clinical implications of potential treatment targets in many different mental disorders in which affective instabilities are a feature.
